# Blood Flow Restriction Training as a Non-Pharmacologic Therapy with Exercise-Induced Hypertension

**DOI:** 10.3390/jcm14134466

**Published:** 2025-06-23

**Authors:** Young-Joo Kim, Ick-Mo Chung, Choung-Hwa Park, Jong-Young Lee

**Affiliations:** 1 School of Sports Science, Sungshin Women’s University, Seoul 02844, Republic of Korea; kyj87@sungshin.ac.kr; 2 Division of Cardiology, Mokdong Hospital, School of Medicine, Ewha Womans University, Seoul 02844, Republic of Korea; ickmo@ewha.ac.kr; 3 Graduate School of Alternative Medicine, Kyonggi University, Seoul 02844, Republic of Korea; 4 Division of Cardiology, Department of Internal Medicine, Kangbuk Samsung Hospital, School of Medicine, Sungkyunkwan University, Seoul 02844, Republic of Korea

**Keywords:** long-distance runner, blood flow restriction, exercise-induced hypertension, graded exercise test

## Abstract

**Background/Objectives:** Long-distance runners with exercise-induced hypertension (EIH) are at increased risk for cardiovascular complications. Although blood flow restriction (BFR) training has shown promise in improving vascular function, hemodynamic response, and cardiorespiratory fitness, its effects in EIH runners remain understudied. This study aimed to evaluate the effects of BFR training on cardiovascular responses and exercise performance in this population as a potential non-pharmacological therapy. **Methods:** Middle-aged male long-distance runners aged 40–65 with peak systolic blood pressure (SBP) ≥ 210 mmHg during graded exercise testing were randomly assigned to either a BFR group (*n* = 18) or a non-BFR control group (*n* = 15) using a computer-generated random sequence. There were no significant differences in baseline characteristics between the groups. Both groups performed aerobic training at 40–60% HRR for 20 min twice weekly for 8 weeks. SBP, diastolic blood pressure (DBP), rate pressure product (RPP), ventilatory threshold (VT), VO_₂max_, and perceived exertion were assessed before and after the intervention at rest, during exercise, and during recovery. **Results:** Compared to the non-BFR group, the BFR group showed statistically significant reductions in resting and maximal SBP and DBP (*p* < 0.05), along with significant increases in VO_₂max_ and VT (*p* < 0.05). During submaximal exercise and post-exercise recovery, SBP and RPP were significantly lower in the BFR group (*p* < 0.05). The reductions in maximal SBP and DBP were significantly greater in the BFR group than in the control group. **Conclusions:** BFR training led to reduced myocardial workload and enhanced cardiovascular efficiency in male runners with EIH. These findings suggest that BFR training may be a viable non-pharmacological therapy for mitigating cardiovascular risks associated with EIH. Future studies should explore the long-term effects of BFR in broader populations and assess its applicability in clinical settings.

## 1. Introduction

Exercise-induced hypertension (EIH) is recognized as a significant risk factor for the increased prevalence and mortality of cardiovascular and cerebrovascular diseases in the general population [1,2]. EIH is defined as an increase in systolic blood pressure of ≥210 mmHg in men and ≥190 mmHg in women during a graded exercise test [3]. While EIH poses health risks in the general population, it appears to be even more prevalent among long-distance runners, particularly those who engage in marathons and ultra-marathons [4]. This is supported by a recent study of 606 long-distance runners, which found that approximately 56% (*n* = 338) met the criteria for EIH. These athletes are also at increased risk for cardiovascular complications, including arrhythmias [4], myocardial hypertrophy [5], and coronary artery disease [6]. More concerningly, emerging evidence links EIH to an elevated risk of exertion-triggered sudden cardiac death, underscoring the need for effective preventive strategies [7]. Recent studies have highlighted blood flow restriction (BFR) training as a promising non-pharmacological intervention for lowering resting blood pressure in hypertensive patients [8]. BFR uses compression bands to restrict blood flow during low-intensity exercise, thereby eliciting physiological effects similar to high-intensity training [9,10].

Beyond its blood pressure-lowering effects, BFR training has also been shown to elicit acute autonomic responses, particularly the activation of the exercise pressor reflex (EPR), due to ischemia-induced metabolic stress. EPR is a neural reflex that increases sympathetic activity and elevates blood pressure during exercise. Although this mechanism has been well documented in both animal and human studies under ischemic conditions [11,12], it was not directly evaluated in the present study, which instead focused on chronic adaptations observed in a non-restricted state following an eight-week BFR intervention.

Runners with exercise-induced hypertension (EIH) have already undergone long-term training; therefore, their blood pressure cannot be improved solely through the same physical activities they have already been performing. To address this, a specialized intervention known as blood flow restriction (BFR) training was implemented over two months to assess changes in hemodynamic responses and cardiorespiratory fitness at rest, during exercise, and throughout the recovery phase, both before and after a graded exercise test. This study is the first to investigate whether BFR training can serve as a non-pharmacological therapeutic alternative for runners with EIH.

## 2. Materials and Methods

### 2.1. Subjects and Study Protocol

Table 1 presents the participants’ data, including physical characteristics, disease, exercise data, and cardiorespiratory fitness.

Runners with exercise-induced hypertension (EIH) were selected based on a maximum systolic blood pressure (MSBP) increase of ≥210 mmHg during the graded exercise test [3]. The participants were divided into two groups: the blood flow restriction group (BFRG, *n* = 18), consisting of EIH runners who participated in BFR (blood flow restriction) training twice a week for 20 min over two months, and the non-BFR group (non-BFRG, *n* = 15), who did not participate in BFR training. There were no significant differences between the two groups in age, height, weight, or BMI in physical characteristics. In terms of exercise data, there were no significant differences between the groups in exercise history, marathons completed, marathon time, exercise time, exercise intensity, or exercise frequency. No meaningful differences in comorbid conditions were detected. No meaningful differences in disease distribution were found between the two groups. In terms of cardiorespiratory fitness, there were no significant differences between the groups in RHR (rest heart rate), RSBP (rest systolic blood pressure), RDBP (rest diastolic blood pressure), MHR (maximum heart rate), MSBP (maximum systolic blood pressure), MDBP (maximum diastolic blood pressure), TET (total exercise time), and VO_2max_.

**Figure 1:**
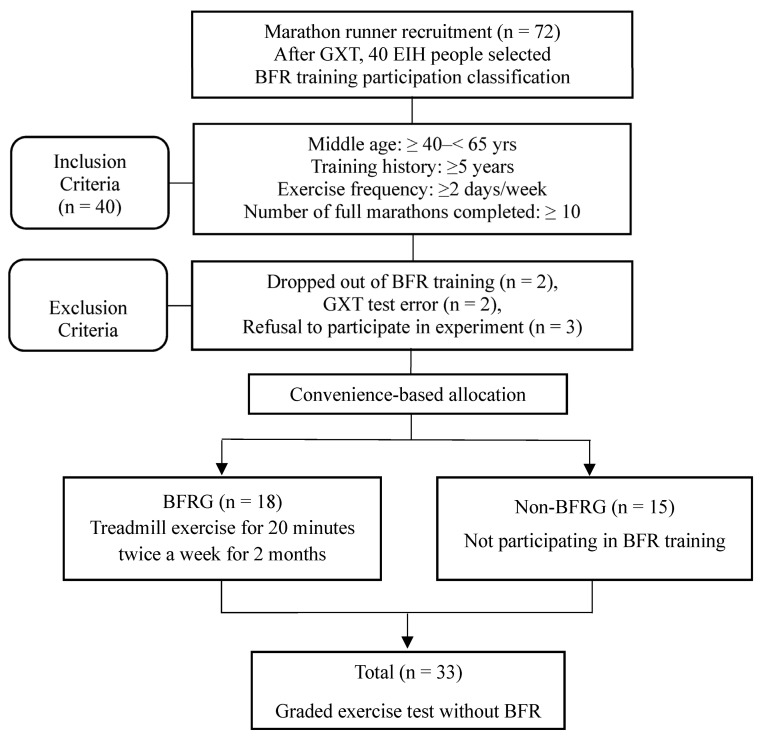
Flow chart of study procedure. GXT: graded exercise testing; BFRG: blood flow restriction group.

The study procedure is presented in Figure 1. Participants were defined as middle-aged males between 40 and 65 years old, with at least five years of exercise history, at least 10 completed marathons, and a minimum exercise frequency of twice per week. Out of the 40 EIH runners initially enrolled, two participants dropped out during the BFR training period, two were excluded due to measurement errors caused by mechanical malfunctions, and three declined to participate in the final experiment. As a result, a total of 33 participants were included in the final analysis (BFRG: 18; non-BFRG: 15). The participants were divided into two groups by convenience-based allocation. Statistical analyses were conducted to compare pre- and post-exercise differences between the two groups. This study was approved by the Institutional Review Board of Korea National Sport University, following the guidelines of the 1975 Declaration of Helsinki (IRB number: 20230921-091).

### 2.2. Graded Exercise Test (GXT)

Participants performed a graded exercise test to measure hemodynamic responses including heart rate (HR), systolic blood pressure (SBP), diastolic blood pressure (DBP), and rate pressure product (RPP) at rest, during each exercise stage, and throughout the recovery phase. Additionally, cardiopulmonary fitness was assessed through respiratory gas analysis, measuring ventilatory threshold and maximal oxygen uptake (VO_₂max_).

A treadmill (T170DE, HP Cosmos, Traunstein, Germany) utilizing the Bruce protocol was used for the graded exercise test. Each stage lasted for three minutes, and at 2 min and 30 s of each stage, participants’ rating of perceived exertion (RPE, Borg scale), blood pressure (Tango+, SunTech, Morrisville, NC, USA), heart rate, and electrocardiogram (ECG) data (CH2000, Cambridge Heart, Tewksbury, MA, USA) were measured (Table 2).

Importantly, to capture real-time hemodynamic responses, SBP, DBP, HR, and RPP were measured during exercise—specifically at 2 min and 30 s into each stage, while participants were actively exercising on the treadmill. These measurements were conducted manually by an experienced examiner using auscultation via a high-performance microphone and headphones for accuracy.

Furthermore, HR, blood pressure, and RPP were continuously monitored and recorded across all phases, including pre-exercise resting state, each exercise stage, and the full 3 min post-exercise recovery period. Respiratory gas analysis (Quark CPET, COSMED, Lavio, Italy) was conducted using a breath-by-breath method at 3 s intervals, with the sampling frequency adjusted to every 30 s starting from Stage 4.

All procedures for the graded exercise test were conducted in accordance with the ACC/AHA guidelines [13].

### 2.3. Blood Flow Restriction Exercise Method

All participants performed low- to moderate-intensity exercise at 40–60% heart rate reserve (HRR), calculated using the Karvonen formula based on data obtained from the graded exercise test. The treadmill exercise was adjusted by modifying the incline and speed accordingly. Participants engaged in the training sessions twice a week for 20 min per session between 6:00 P.M. and 9:00 P.M. for eight weeks. The training program included both Cycle mode and Constant mode functions. Cycle mode consisted of three intensity levels: Low SKU (150–220 mmHg), Medium SKU (230–300 mmHg), and High SKU (330–400 mmHg). In this study, a fixed stepwise occlusion pressure protocol (SKU, standard Kaatsu unit) was applied. Although individual occlusion pressure calibration using Doppler ultrasound was not conducted, the SKU protocol has been widely used in previous studies and was chosen to ensure consistency and feasibility across participants. It is applied to the proximal thigh, and at each intensity, pressure is maintained for 30 s before being released for 5 s. The occlusion pressure increases by 10 mmHg with each cycle, delivering eight stimulations per cycle. For example, in the case of Low SKU (150–220 mmHg), an initial pressure of 150 mmHg is applied for 30 s, followed by a 5 s release. The pressure then automatically increases to 160 mmHg, which is maintained for another 30 s before a 5 s release. This cycle continues until the pressure reaches 220 mmHg, allowing participants to experience a total of eight pressure applications. This method promotes intermittent blood flow, making it easier to perform exercises compared to the Constant mode, which continuously restricts blood flow. The Constant mode maintains a preset pressure continuously for a maximum duration of 20 min. To monitor blood flow, the area above the knee is firmly pressed with the thumb and then released. If a color change from pale white to reddish (indicating blood reperfusion during exercise) does not occur within 3 s, this signals impaired blood circulation. In such cases, the pressure intensity was reduced, or the training was stopped. To ensure the proper application of BFR training, all supervision and measurements were conducted by certified Kaatsu specialists who had obtained official qualifications issued by the Kaatsu organization (Table 3).

For the exercise program, the first week served as an adaptation period for BFR training. Participants exercised on a treadmill at 50% HRR while wearing a band on the upper thigh. The Cycle mode (Low SKU) was applied, delivering continuous pressure for 30 s followed by a 5 s release, repeated for a total of 20 min. The second session was conducted by applying the Cycle mode (Medium SKU) for 10 min, followed by a transition to the Constant mode at 250 mmHg, which was maintained for an additional 10 min. The protocol remained the same as in the second week from the third to the eighth week, except that the Cycle mode was applied only once. This was followed by continuous blood flow restriction for 15 min, maintaining an intensity at 60% of HRR.

### 2.4. Statistical Analysis

Statistical analyses were performed using SPSS Statistics version 21. All data are presented as means ± standard deviation. Group differences in physical characteristics, exercise-related variables, and cardiorespiratory fitness were assessed using independent samples *t*-tests. To examine the main effects of time, group, and their interaction (time × group), a two-way repeated measures analysis of variance (ANOVA) was conducted. Effect sizes for each ANOVA result were reported using partial eta squared (η²ₚ), with values of 0.01, 0.06, and 0.14 interpreted as small, medium, and large effects, respectively, according to established benchmarks [14]. The normality of the data distribution was assessed using the Shapiro–Wilk test prior to the application of parametric tests. When a significant interaction effect was observed, paired *t*-tests were used for within-group comparisons (pre- vs. post-intervention), and independent *t*-tests were applied for between-group comparisons at each time point. Missing data due to participant dropout or equipment malfunction were excluded from the relevant analyses using listwise deletion. The level of statistical significance was set at *p* < 0.05.

## 3. Results

Table 4 presents the changes observed at rest and during maximal exercise testing before and after BFR training. There was no significant interaction effect for resting heart rate (RHR) and resting systolic blood pressure (RSBP). Resting diastolic blood pressure (RDBP) remained unchanged in the BFR group (BFRG) but increased significantly in the non-BFR group (non-BFRG) (*p* < 0.05).

Table 5 presents the changes observed during submaximal exercise testing before and after BFR training. In Stage 1, among heart rate (HR), systolic blood pressure (SBP), diastolic blood pressure (DBP), and rating of perceived exertion (RPE), only SBP exhibited a significant reduction in the BFR group (BFRG) (*p* < 0.05), whereas no significant change was observed in the non-BFR group (non-BFRG), indicating a significant interaction effect (*p* < 0.05). In Stage 2, while HR and RPE showed no significant interaction effects, maximal systolic blood pressure (MSBP) and DBP significantly decreased in the BFRG (*p* < 0.05), whereas no significant changes were observed in the non-BFRG, demonstrating a significant interaction effect (*p* < 0.05). In Stage 3, HR remained unchanged, but both SBP and DBP significantly decreased in the BFRG (*p* < 0.05), while no significant differences were observed in the non-BFRG, resulting in a significant interaction effect (*p* < 0.05). Additionally, RPE did not significantly change in the BFRG, but a significant increase was observed in the non-BFRG (*p* < 0.05), confirming a significant interaction effect (*p* < 0.05).

Table 6 presents the hemodynamic changes during the recovery period before and after BFR training. From 1 min to 3 min of recovery, no significant interaction effects were observed for heart rate (HR) and diastolic blood pressure (DBP). However, SBP significantly decreased in the BFRG, whereas no change was observed in the non-BFRG, confirming a significant interaction effect (*p* < 0.05).

**Figure 2:**
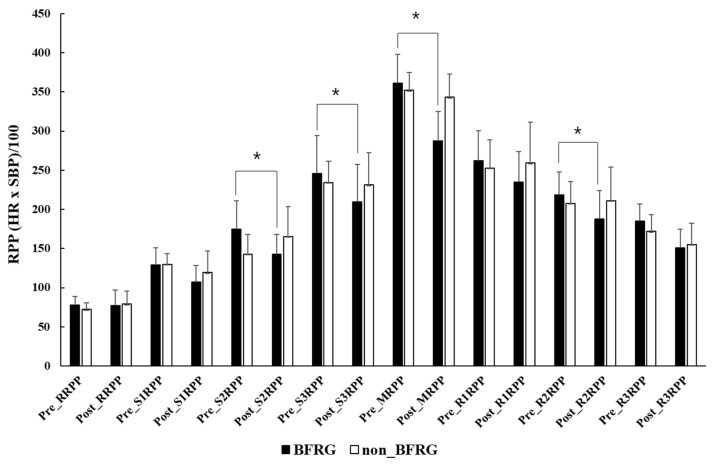
Changes in RPP in GXT before and after BFR training. RRPP: rest rate pressure product; S: stage; MRPP: maximum rate pressure product; R: recovery; BFRG: blood flow restriction group; *: significant difference between pre- and post-exercise at *p* < 0.05.

Figure 2 shows the changes in rate pressure product (RPP) during the graded exercise tests, comparing measurements before and after BFR training (values in parentheses indicate pre- vs. post-training results). The resting RPP (RRPP) was recorded as 77.9 ± 11.1 vs. 77.5 ± 19.8 in the BFR group (BFRG) and 72.3 ± 8.3 vs. 79.1 ± 16.3 in the non-BFR group (non-BFRG). At Stage 1, RPP was 128.8 ± 22.3 for the BFRG compared to 107.4 ± 21.2 for the non-BFRG, while Stage 2 RPP was 175.1 ± 35.8 vs. 142.8 ± 25.5 in the BFRG and 142.8 ± 25.5 vs. 165.5 ± 38.2 in the non-BFRG. For Stage 3, RPP was 245.8 ± 48.7 vs. 209.8 ± 47.9 in the BFRG and 234.4 ± 27.4 vs. 231.2 ± 41.2 in the non-BFRG. The maximal RPP (MRPP) was 361.1 ± 37.1 vs. 287.3 ± 37.4 in the BFRG and 352.0 ± 22.5 vs. 347.5 ± 21.6 in the non-BFRG. During the recovery phase, RPP at 1 min (R1RPP) was 261.9 ± 38.4 vs. 234.9 ± 39.1 in the BFRG and 252.8 ± 36.1 vs. 259.2 ± 51.9 in the non-BFRG, RPP at 2 min (R2RPP) was 218.6 ± 29.1 vs. 187.9 ± 35.8 in the BFRG and 207.9 ± 27.6 vs. 211.0 ± 42.7 in the non-BFRG, and RPP at 3 min (R3RPP) was 184.9 ± 21.9 vs. 151.2 ± 23.4 in the BFRG and 172.0 ± 21.5 vs. 155.3 ± 27.0 in the non-BFRG. Among these, Stage 2 RPP (S2RPP), Stage 3 RPP (S3RPP), MRPP, and RPP at 2 min of recovery (R2RPP) showed significantly greater reductions in the BFRG compared to the non-BFRG (*p* < 0.05), indicating a significant interaction effect (*p* < 0.05). The effect size (partial eta squared η²ₚ) values for time, group, and interaction effects across each stage were as follows: RRPP (T: 0.044; G: I:0.007; 0.054), S1RPP (T: 0.401; G: 0.030; I: 0.072), S2RPP (T: 0.876; G: 0.018; I: 0.243), S3RPP (T: 0.235; G: 0.004; I: 0.177), MRPP (T: 0.592; G: 0.158, I: 0.474), R1RPP (T:0.035; G: 0.016; I: 0.086), R2RPP (T:0.094; G:0.014; I:0.136), and R3RPP (T: 0.481; G: 0.014; I: 0.095).

**Figure 3:**
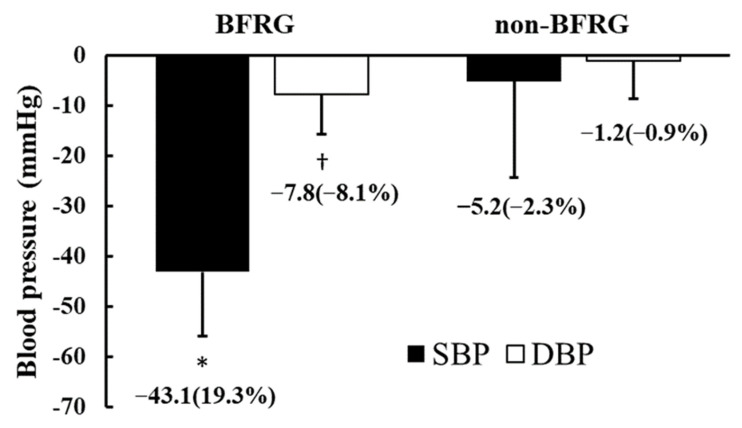
Changes in maximal blood pressure in response to BFR training. BFRG: blood flow restriction group; SBP: systolic blood pressure; DBP: diastolic blood pressure. * Significant difference in SBP between BFRG and non-BFRG (*p* < 0.05); †: Significant difference in DBP between BFRG and non-BFRG (*p* < 0.05).

Figure 3 illustrates the reductions and percentage changes in maximal systolic blood pressure (MSBP) and maximal diastolic blood pressure (MDBP) before and after BFR training. The absolute difference in MSBP before and after exercise was −43.1 ± 19.2 mmHg in the BFR group (BFRG), with a percentage change of −19.3 ± 8.3%, whereas in the non-BFR group (non-BFRG), the absolute difference was −5.2 ± 12.9 mmHg, with a percentage change of −2.3 ± 5.8%. Both the absolute difference and percentage change in MSBP were significantly greater in the BFRG than in the non-BFRG (*p* < 0.05). Similarly, for MDBP, the absolute difference before and after exercise was −7.8 ± 7.9 mmHg in the BFRG, with a percentage change of −8.1 ± 8.5%, while in the non-BFRG, the absolute difference was −1.2 ± 7.5 mmHg, with a percentage change of −0.9 ± 8.0%. The absolute difference and percentage change in MDBP showed significantly greater reductions in the BFRG compared to the non-BFRG (*p* < 0.05).

## 4. Discussion

This study is the first to investigate the effects of a two-month aerobic exercise program incorporating blood flow restriction (BFR) training in long-distance runners with exercise-induced hypertension (EIH). The results demonstrated that BFR training contributed to favorable cardiovascular adaptations, including reductions in both resting and exercise-induced blood pressure. Notably, BFR training led to significant improvements in maximal systolic and diastolic blood pressure responses during graded exercise testing, while no such benefits were observed in the non-BFR group. These findings suggest that BFR training may help mitigate excessive blood pressure responses during exercise, a hallmark of EIH, and promote hemodynamic stability in endurance athletes (Figure 3).

However, the relationship between BFR training and changes in resting blood pressure has not been firmly established, and research on blood pressure responses during exercise remains extremely limited. Most existing studies have primarily focused on the finding that even a single acute bout of exercise with conventional BFR application can lead to a temporary reduction in resting blood pressure [15,16]. Zhao et al. [8]. examined the effects of long-term BFR training in hypertensive patients, applying BFR to the proximal lower limbs three times per week for 12 weeks while performing five sets of 20 repetitions of leg extensions. Their findings demonstrated a reduction in systolic blood pressure and improvements in autonomic nervous system regulation.

In this study, the significant reduction in maximal systolic and diastolic blood pressure observed during the graded exercise test suggests that BFR training may contribute to improved vascular function and hemodynamic stability.

Although VEGF expression was not directly measured in the present study, previous research has suggested that BFR training under hypoxic conditions can upregulate vascular endothelial growth factor (VEGF) and stimulate angiogenesis in the lower limbs [17,18]. This mechanism likely contributes to improved endothelial function and peripheral blood circulation [19], thereby reducing afterload during exercise. These effects were also evident in the significant reductions in blood pressure during submaximal exercise and the recovery period (Table 5 and Table 6), while myocardial workload demonstrated consistent improvements across all conditions (Figure 2). A reduction in RPP reflects a decreased myocardial oxygen demand during exercise, which is particularly relevant in populations with elevated cardiovascular risk, such as those with EIH. Lower RPP values are associated with improved cardiac efficiency and reduced afterload, ultimately lowering the risk of myocardial ischemia during high-intensity activity [20]. Excessive blood pressure elevation during exercise leads to an increased myocardial workload, which, in patients with angina, raises the risk of myocardial ischemia, potentially resulting in chest pain, acute myocardial infarction, or sudden cardiac death [21]. Long-distance runners with exercise-induced hypertension (EIH) experience elevated blood pressure during exercise, which has been associated with post-marathon increases in cardiac biomarker levels such as cardiac troponin I (cTnI) and N-terminal pro-brain natriuretic peptide (NT-proBNP), markers of myocardial injury and volume overload, respectively [22].

Runners with exercise-induced hypertension (EIH) have been reported to exhibit a higher incidence of arrhythmias [4], left ventricular hypertrophy [5], and an increased prevalence of coronary plaque formation [6] due to chronically elevated blood pressure during exercise. Given these conditions, runners with EIH may face a relatively higher risk of sudden cardiac death during exercise [7]. As exercise-induced hypertension (EIH) is a significant risk factor for increased cardiovascular and cerebrovascular disease incidence and mortality, research has primarily focused on pharmacological interventions [7]. Among these, beta-blockers (BBs) have been reported to be more effective in reducing blood pressure during exercise compared to angiotensin-converting enzyme inhibitors (ACEis), diuretics, and calcium-channel blockers (CCBs) [23]. Additionally, angiotensin receptor blockers (ARBs) have been reported to lower maximal systolic blood pressure (SBP) by up to 33 mmHg [24]. Kim et al. [25]. reported that angiotensin-converting enzyme inhibitors (ACEIs) are recommended for runners with exercise-induced hypertension (EIH), as they exhibit heightened activation of angiotensin II within the renin–angiotensin–aldosterone system (RAAS). Although various risks associated with EIH have been documented in both the general population and endurance athletes, and several pharmacological treatment options have been proposed, no official guidelines have been established to date. Given this lack of standardized recommendations, BFR training may serve as a promising non-pharmacological intervention, potentially offering an effective alternative that does not require a physician’s prescription.

Another significant effect of BFR training is its ability to enhance maximal oxygen consumption (VO_₂max_) and ventilatory threshold (VT) (Table 4), which are key indicators of maximal exercise capacity—even in experienced athletes who consistently engage in regular training. Notably, improvements in VO_₂max_, a primary marker of cardiorespiratory fitness, have been independently associated with lower all-cause and cardiovascular mortality [26]. Even modest increases in VO_₂max_ have been shown to significantly reduce the risk of adverse cardiovascular outcomes [27], highlighting the clinical relevance of enhancing maximal exercise capacity through BFR training.

Barjaste et al. [28]. reported that even a single session of acute BFR training combined with aerobic exercise activates signaling pathways associated with protein expression, mitochondrial biogenesis, and angiogenesis induced by ischemia in the lower limbs. When BFR training is performed regularly and becomes chronic, it has been shown to increase VO_₂max_ in sprint athletes, endurance athletes, and young healthy individuals [29,30,31]. However, in this study, the extent to which the effects of a two-month BFR aerobic training program persist over time remains unclear. Further research is required to establish guidelines for the sustained antihypertensive effects of BFR in EIH and to determine whether resistance exercise, rather than aerobic exercise, can yield similar benefits.

This study has several limitations. First, it was not possible to fully control for individual lifestyle factors such as physical activity levels, alcohol consumption, diet, and sleep, which may have influenced the training responses.

Second, the sample size was relatively small, and all participants were middle-aged males, limiting the generalizability of the findings. Sex- and age-specific cardiovascular responses, particularly in relation to autonomic regulation and vascular reactivity, may differ across populations and should be examined in future studies.

Third, although the participants did not present with known cardiovascular disease, the presence of subclinical or undetected conditions cannot be entirely ruled out.

Fourth, this study did not include any real-time hemodynamic assessments during the BFR exercise sessions. As such, we could not evaluate acute cardiovascular responses such as the exercise pressor reflex or metaboreflex, which may be activated during ischemic conditions. Future studies should incorporate continuous monitoring during BFR exercise to better assess its acute safety and physiological effects in runners with EIH.

Fifth, we did not individualize the BFR stimulus using arterial occlusion pressure (AOP) percentages. A fixed pressure protocol (Cycle mode and Constant mode) was used, which may have resulted in variable occlusion levels among participants. This limits the comparability to AOP-standardized protocols and may have influenced individual responses.

Sixth, real-time physiological and molecular assessments (e.g., catecholamines, VEGF, RAAS markers) during BFR exercise were not performed, and as such, the acute mechanistic underpinnings of the observed benefits remain speculative.

Seventh, while SEVR (subendocardial viability ratio) would offer insight into myocardial oxygen supply–demand balance, our study did not incorporate continuous blood pressure monitoring, and thus SEVR could not be calculated

Finally, the long-term effects of the intervention beyond the two-month training period were not assessed, and the observed benefits may be temporary.

## 5. Conclusions

BFR training in runners with EIH has been shown to reduce blood pressure at rest, submaximal, maximal, and recovery stages while simultaneously enhancing cardiorespiratory fitness and improving overall myocardial workload, supporting its potential application as a non-pharmacological alternative therapy. Future studies should examine the long-term effects of BFR training and investigate its efficacy and safety across diverse populations, including female athletes and individuals with varying levels of cardiovascular risk.

## Figures and Tables

**Table 1:** Characteristics of study participant demographics.

Variable	BFRG (*n* = 18)	Non-BFRG (*n* = 15)	*p*-Value
Physical characteristics			
Age (years)	57.2 ± 5.2	59.1 ± 6.6	0.379
Height (cm)	172.0 ± 6.6	169.6 ± 4.1	0.220
Weight (kg)	67.7 ± 7.2	66.2 ± 7.5	0.559
BMI (m^2^/kg)	22.8 ± 2.1	22.9 ± 2.3	0.923
Disease			
Hypertension	5 (27.8%)	3 (20.0%)	0.604
Dyslipidemia	3 (16.7%)	1 (6.7%)	0.381
Diabetes + hypertension	0 (0%)	2 (13.3%)	0.110
Hypertension + dyslipidemia	2 (11.1)	1 (6.7%)	0.658
Exercise data			
Exercise history (yrs)	17.2 ± 6.5	19.1 ± 5.7	0.384
Marathon completed (numbers)	72.5 ± 49.9	56.4 ± 36.1	0.306
Marathon time (min)	233.7 ± 38.1	234.2 ± 39.8	0.972
Exercise time (min/day)	74.4 ± 20.9	89.3 ± 31.2	0.113
Exercise intensity (Borg’s scale)	14.1 ± 1.2	14.7 ± 1.0	0.181
Exercise frequency (weeks)	4.1 ± 1.6	4.2 ± 1.5	0.787
Cardiorespiratory fitness			
RHR (beats/min)	61.3 ± 8.7	58.0 ± 4.6	0.194
RSBP (mmHg)	127.5 ± 12.1	124.6 ± 10.4	0.483
RDBP (mmHg)	82.5 ± 9.5	78.3 ± 5.2	0.143
MHR (beats/min)	163.3 ± 13.6	162.9 ± 7.4	0.456
MSBP (mmHg)	221.7 ± 7.3	217.5 ± 5.7	0.112
MDBP (mmHg)	94.0 ± 8.0	94.2 ± 8.4	0.926
TET (s)	788.3 ± 138.0	784.6 ± 84.2	0.929
Ventilatory threshold (%)	40.4 ± 10.5	40.4 ± 7.6	0.984
VO_2max_ (mL/kg/min)	46.4 ± 9.0	46.6 ± 6.6	0.941

Values presented as mean ± standard deviation; BFRG: blood flow restriction group; BMI: body mass index; RHR: rest heart rate; SBP: systolic blood pressure; DBP: diastolic blood pressure; MHR: maximum heart rate; MSBP: maximum systolic blood pressure; MDBP; maximum diastolic blood pressure; TET: total exercise time.

**Table 2:** GXT method based on Bruce protocol.

Bruce Protocol			Rest	Exercise	Recovery
Stage	Speed (mph)	Grade (Slope%)			
1	1.7	10	After a sufficient 5 min rest, HR, SBP, DBP, and RPP were measured.	RPE, SBP, DBP, HR, VO_2_, and ECG were measured at 2 min and 30 s of each GXT stage. From Stage 4 onward, measurements were recorded every 30 s. For precise BP measurement, a high-sensitivity microphone was attached to the brachioradial artery to detect Korotkoff sounds.	After the completion of the test, HR, BP, RPP, and ECG were measured at 1 min intervals for up to 3 min, after which the test was terminated.
2	2.5	12			
3	3.4	14			
4	4.2	16			
5	5.0	18			
6	5.5	20			
7	6.0	22			
Recovery	1.7	0			

GXT: graded exercise test; Reco: recovery; RPE: rating of perceived exertion; SBP: systolic blood pressure; DBP: diastolic blood pressure; HR: heart rate; ECG: electrocardiography.

**Table 3:** The 8-Week BFR training protocol.

Weeks	Intensity	Frequency/Time	Method
Week 1	50% HRR	Two a week/ 20 min	Cycle mode (Low SKU, 150–220 mmHg): Begins with 150 mmHg pressure for 30 s followed by 5 s release; pressure increases by 10 mmHg per stage; repeated for 30 s on/5 s off up to 220 mmHg across 8 stages.
Week 2	50% HRR	Two a week/ 20 min	Cycle mode (Low SKU, 150–220 mmHg): Begins with 150 mmHg pressure for 30 s followed by 5 s release; pressure increases by 10 mmHg per stage; repeated as 30 s on/5 s off over 8 stages up to 220 mmHg, applied for a total of 10 min. Then switched to Constant mode (250 mmHg) for 10 min during exercise.
Week 3~8	60% HRR	Two a week/ 20 min	Cycle mode (Medium SKU, 230–300 mmHg): One cycle of Stages 1–8 was applied, followed by 15 min of Constant mode at 250 mmHg.

**Table 4:** Changes in resting and maximal exercise stress test before and after BFR.

Variable	Group	Pre	Post	*p*-Value (Partial Eta Squared)
HR_rest_ (beats/min)	BFRG	61.3 ± 8.7	64.4 ± 13.3	T: 0.019 (0.164) G: 0.435 (0.020) I: 0.580 (0.010)
	non-BFRG	58.0 ± 4.6	62.9 ± 10.2 *	
SBP_rest_ (mmHg)	BFRG	127.5 ± 12.1	119.8 ± 9.1 *	T: 0.154 (0.064) G: 0.676 (0.006) I: 0.091 (0.089)
	non-BFRG	124.6 ± 10.4	125.3 ± 12.7	
DBP_rest_ (mmHg)	BFRG	82.5 ± 9.5	82.5 ± 9.2	T: 0.016 (0.174) G: 0.990 (0.000) I: 0.017 (0.170)
	non-BFRG	78.3 ± 5.2	86.6 ± 5.5 *	
HR_max_ (beats/min)	BFRG	163.3 ± 13.6	160.7 ± 18.5	T: 0.959 (0.000) G: 0.612 (0.008) I: 0.197 (0.053)
	non-BFRG	162.9 ± 7.4	165.4 ± 7.2	
SBP_max_ (mmHg)	BFRG	221.7 ± 7.3	178.6 ± 17.6 *§	T: <0.001 (0.666) G: <0.001 (0.313) I: <0.001 (0.534)
	non-BFRG	217.5 ± 5.7	212.3 ± 13.9	
DBP_max_ (mmHg)	BFRG	94.0 ± 8.0	86.1 ± 9.0 *§	T: 0.002 (0.267) G: 0.171 (0.060) I: 0.021 (0.160)
	non-BFRG	94.2 ± 8.4	93.0 ± 7.5	
TET (s)	BFRG	788.3 ± 138.0	808.3 ± 125.5	T: 0.274 (0.038) G: 0.820 (0.002) I: 0.713 (0.004)
	non-BFRG	784.6 ± 84.2	794.6 ± 92.9	
VT	BFRG	40.4 ± 10.5	46.0 ± 7.1 *§	T: 0.009 (0.203) G: 0.310 (0.033) I: 0.011 (0.190)
	non-BFRG	40.4 ± 7.6	40.4 ± 9.2	
VO_2max_ (ml/kg/min)	BFRG	46.4 ± 9.0	49.9 ± 7.8 *	T: 0.121 (0.121) G: 0.386 (0.024) I: <0.001 (0.374)
	non-BFRG	46.6 ± 6.6	45.0 ± 6.8	

Values presented as means ± standard deviation; BFRG: blood flow restriction group; HR: heart rate; SBP: systolic blood pressure; DBP: diastolic blood pressure; TET: total exercise time; VT: ventilation threshold; T: time; G: group; I: interaction (time x group); *: significant difference between pre- and post-exercise values at *p* < 0.05; §: significant difference between BFRG and non-BFRG at *p* < 0.05.

**Table 5:** Hemodynamic changes in submaximal exercise stress test before and after BFR.

	Variable	Group	Pre	Post	*p*-Value (Partial Eta Squared)
Stage 1	HR	BFRG	89.1 ± 12.4	83.8 ± 13.9 *	T: 0.006 (0.218) G: 0.473 (0.017) I: 0.917 (0.000)
		non-BFRG	92.3 ± 10.2	86.6 ± 14.1	
	SBP	BFRG	144.2 ± 11.5	128.0 ± 10.3 *	T: <0.001 (0.386) G: 0.397 (0.023) I: 0.012 (0.189)
		non-BFRG	141.2 ± 11.8	137.3 ± 15.6	
	DBP	BFRG	82.8 ± 8.3	78.2 ± 10.8 *	T: 0.082 (0.094) G: 0.435 (0.055) I:0.189 (0.020)
		non-BFRG	83.0 ± 5.7	82.3 ± 9.0	
	RPE	BFRG	8.0 ± 1.4	7.1 ± 1.1	T: 0.043 (0.126) G: 0.735 (0.004) I: 0.345 (0.029)
		non-BFRG	7.6 ± 1.4	7.3 ± 0.7	
Stage 2	HR	BFRG	106.8 ± 14.3	103.8 ± 13.9	T: 0.317 (0.037) G: 0.908 (0.000) I: 0.519 (0.014)
		non-BFRG	105.2 ± 9.5	104.3 ± 14.1	
	SBP	BFRG	163.0 ± 16.2	137.2 ± 12.0 *§	T: <0.001 (0.416) G: 0.086 (0.092) I: <0.001 (0.367)
		non-BFRG	158.6 ± 12.3	157.3 ± 19.2	
	DBP	BFRG	85.8 ± 7.7	77.9 ± 8.3 *§	T: 0.003 (0.252) G: 0.514 (0.014) I: 0.001 (0.292)
		non-BFRG	83.2 ± 6.3	83.6 ± 7.1	
	RPE	BFRG	9.8 ± 1.9	9.8 ± 2.2	T: 0.286 (0.037) G: 0.286 (0.000) I: 1.000 (0.037)
		non-BFRG	9.4 ± 2.0	10.2 ± 1.1	
Stage 3	HR	BFRG	133.3 ± 16.5	133.0 ± 17.3	T: 0.897 (0.001) G: 0.707 (0.005) I: 0.964 (0.000)
		non-BFRG	131.4 ± 10.8	131.1 ± 9.1	
	SBP	BFRG	183.1 ± 16.6	156.5 ± 19.3 *§	T: <0.001(0.340) G: 0.225 (0.047) I: 0.002 (0.262)
		non-BFRG	178.1 ± 13.2	175.6 ± 26.3	
	DBP	BFRG	89.1 ± 8.6	79.7 ± 8.6 *§	T: 0.001 (0.295) G: 0.496 (0.015) I: 0.002 (0.278)
		non-BFRG	86.2 ± 6.4	86.0 ± 7.1	
	RPE	BFRG	12.8 ± 2.3	12.2 ± 2.3	T: 0.449 (0.019) G: 0.805 (0.002) I: 0.002 (0.263)
		non-BFRG	11.8 ± 1.4	12.9 ± 1.0 *	

Values presented as mean ± standard deviation; BFRG: blood flow restriction group; HR: heart rate; SBP: systolic blood pressure; DBP: diastolic blood pressure; RPE: rating of perceived exertion; T: time; G: group; I: interaction (time x group); *: significant difference between pre- and post-exercise at *p* < 0.05; §: significant difference between BFRG and non-BFRG at *p* < 0.05.

**Table 6:** Hemodynamic changes during recovery before and after BFR.

	Variable	Group	Pre	Post	*p*-Value (Partial Eta Squared)
Recovery 1 min	HR	BFRG	123.2 ± 14.8	134.6 ± 21.8	T: <0.001 (0.327) G: 0.485 (0.016) I: 0.415 (0.022)
		non-BFRG	124.1 ± 17.8	141.6 ± 21.2 *	
	SBP	BFRG	212.1 ± 12.0	175.1 ± 17.2 *	T: <0.001 (0.633) G: 0.974 (0.000) I: 0.041 (0.128)
		non-BFRG	203.9 ± 11.0	183.6 ± 27.2 *	
	DBP	BFRG	93.3 ± 8.8	83.6 ± 8.0 *	T: <0.001 (0.535) G: 0.536 (0.012) I: 0.286 (0.037)
		non-BFRG	93.4 ± 7.5	86.6 ± 6.9 *	
Recovery 2 min	HR	BFRG	109.6 ± 13.2	112.7 ± 17.5	T: 0.108 (0.081) G: 0.699 (0.005) I: 0.612 (0.008)
		non-BFRG	110.0 ± 14.6	116.0 ± 17.6	
	SBP	BFRG	199.5 ± 13.2 §	166.3 ± 17.9 *§	T: <0.001 (0.515) G: 0.608 (0.009) I: <0.001 (0.307)
		non-BFRG	189.1 ± 11.2	182.0 ± 25.4	
	DBP	BFRG	91.1 ± 9.4	83.6 ± 7.8 *	T: 0.004 (0.244) G: 0.600 (0.009) I: 0.038 (0.132)
		non-BFRG	89.4 ± 7.1	88.0 ± 7.7	
Recovery 3 min	HR	BFRG	99.7 ± 11.8	100.7 ± 14.6	T: 0.572 (0.572) G: 0.902 (0.001) I: 0.937 (0.000)
		non-BFRG	99.0 ± 13.6	100.4 ± 16.0	
	SBP	BFRG	185.7 ± 10.9 §	150.3 ± 15.1 *	T: <0.001 (0.761) G: 0.509 (0.014) I: 0.006 (0.219)
		non-BFRG	174.5 ± 14.6	155.3 ± 20.3 *	
	DBP	BFRG	88.8 ± 9.1	82.9 ± 7.0 *	T: <0.010 (0.196) G: 0.656 (0.006) I: 0.163 (0.062)
		non-BFRG	85.7 ± 6.4	83.8 ± 8.9	

Values presented as mean ± standard deviation; BFRG: blood flow restriction group; HR: heart rate; SBP: systolic blood pressure; DBP: diastolic blood pressure; T: time; G: group; I: interaction (time x group); *: significant difference between pre- and post-exercise at *p* < 0.05; §: significant difference between BFRG and non-BFRG at *p* < 0.05.

## Data Availability

The datasets presented in this article are not readily available because they are part of an ongoing study involving extended follow-up analyses. Requests to access the datasets should be directed to Jong-Young Lee (jyleeheart@naver.com).
